# Sediment biomarkers elucidate the Holocene ontogeny of a shallow lake

**DOI:** 10.1371/journal.pone.0191073

**Published:** 2018-01-11

**Authors:** T. E. Arnold, W. F. Kenney, J. H. Curtis, T. S. Bianchi, M. Brenner

**Affiliations:** 1 Department of Geological Sciences, University of Florida, Gainesville, Florida, United States of America; 2 Land Use and Environmental Change Institute, University of Florida, Gainesville, Florida, United States of America; The University of Hong Kong, HONG KONG

## Abstract

We carried out geochemical analyses on a sediment core from Lake Harris, Florida (USA) to identify sources of organic matter to the sediment throughout the Holocene, and relate changes in those sources to shifts in past climate and environmental conditions. We hypothesized that the sources of organic matter changed in response to regional hydrologic shifts following de-glaciation, and to human population expansion in the state during the 20^th^ century. Hydroclimate shifts in Florida were related to: 1) a steady rise in relative sea level and the fresh water table that began in the early Holocene, 2) wetland formation and expansion ca. 5,000 cal yrs BP, and 3) the onset of the modern El Niño (ENSO) cycle ~3,000 cal yrs BP. Stratigraphic changes in sediment variables from Lake Harris reflect each of these hydroclimate periods. Early in the Holocene, Lake Harris was a marsh-like system in a relatively dry, open-prairie environment. Organic sediments deposited at that time were derived largely from terrestrial sources, as inferred from high TOC/TN ratios, a dominance of longer-chain of *n*-alkanes (*n*-C_29-31_), relatively negative organic carbon isotope values (δ^13^C_TOC_), and low biogenic silica concentrations. In the middle Holocene, a positive shift in δ^13^C_TOC_ coincided with the onset of wetter conditions in Florida. Submerged macrophyte biomarkers (*n*-C_21-23_) dominated, and during that period bulk organic carbon isotope values were most similar to δ^13^C values of mid-chain-length *n*-alkanes. In the late Holocene, δ^13^C_TOC_ values declined, CaCO_3_ levels decreased to trace amounts, organic carbon concentrations increased and diatom biogenic silica concentrations increased from 10 to 120 mg g^-1^. Around 2,900 cal yrs BP, the effects of ENSO intensified and many Florida lakes deepened to their current limnetic state. Concentrations of algal and cyanobacterial biomarkers in the Lake Harris core increased by orders of magnitude after about AD 1940, in response to human-induced eutrophication, an inference supported by values of δ^15^N that fluctuate around zero.

## Introduction

Lakes are critical components of the terrestrial carbon cycle. Their sediment contains an archived history of changes in organic carbon sources from the watershed (allochthonous) and water column (autochthonous). The sediments of lakes, reservoirs, and wetlands constitute a pool of organic carbon that is sequestered at a rate (0.07 Pg C yr^-1^) estimated to exceed the rate in the ocean by a factor of three [1, 2]. Analyses of organic carbon in a lacustrine sediment archive can be used to infer past environmental changes that occurred during the lake’s existence. In Florida, paleolimnological reconstructions based on organic biomarkers were used to document ecological succession [3] and hydrologic changes [4] during the transition from the last glacial period to the present.

Paleolimnological inferences regarding past climate and environment routinely employ total organic carbon/total nitrogen (TOC/TN) ratios and stable isotope values of total organic carbon (δ^13^C_TOC_) to identify sources of organic matter in the lake sediments [5, 6]. Low TOC/TN ratios in algae arise from their lack of carbohydrate-rich structural components [7]. Moreover, the extreme differences in the range of TOC/TN values in vascular (>20) versus non-vascular plants (4–10) have been used to estimate the relative contributions from these organic carbon sources to sediments.

Similarly, the δ^13^C_TOC_ of bulk lake sediment can be interpreted as reflecting a mixture of values from various end-member groups. The δ^13^C values of higher plant and algal organic matter are governed primarily by the isotope value of the carbon substrate used for photosynthesis (atmospheric CO_2_ or dissolved HCO_3_^-^) and the enzymatic fractionations associated with fixing that carbon (i.e. ribulose 1,5 bisphosphate [RuBP]) [8]. Most terrestrial plants assimilate carbon through one of two photosynthetic pathways (C_3_ or C_4_) and the δ^13^C values of C_3_ plants (most trees, shrubs and herbs) differ from those of C_4_ plants (tropical grasses) [9]. Algae use the C_3_ pathway for photosynthesis, but in high-pH aquatic systems they typically display larger δ^13^C values because dissolved HCO_3_^-^ is enriched in ^13^C relative to CO_2(*aq)*_ by ~9‰ [8].

Recent studies, however, suggest that interpretation of organic carbon sources in lake sediments from TOC/TN ratios alone must be undertaken with caution. For instance, Cloern et al. [10] showed that TOC/TN ratios vary considerably at the species level. It has also been noted that TOC/TN can be greater in N-limited systems [11], and lower in systems where there is selective degradation of labile carbon [12]. Similarly, the interpretation of organic matter provenance from δ^13^C_TOC_ can be equivocal. In lake sediments, stratigraphic shifts toward more positive δ^13^C values have been explained in multiple ways, such as reflecting greater input from algal material [13], increased autotrophic carbon fixation [14, 15], and a transition from a C_3_ to a C_4_ terrestrial plant community in the watershed [16].

An alternative to analysis of bulk organic matter in lake sediments is the application of compound-specific isotope measurements of molecular biomarkers to track the source and history of sediment organic matter. Biomarker studies have enabled reconstructions of past hydrological changes across millennial time scales. For example, carbon isotope values of lipid compounds in sediments from Mud Lake, Florida were used to document vegetation changes associated with Holocene climate shifts [17]. The authors found that shifts in alkane biomarkers corresponded to fluctuations in the regional water table that were caused by hydroclimate changes in the early Holocene. Sediment cores from other Florida lakes showed that changes recorded in the carbon isotopes of the *n*-alkanes were consistent with pollen-based environmental reconstructions [3].

A recent study of Lake Harris, Florida, USA, used multiple geochemical proxies from a sediment core dated to ~10,000 cal yrs BP, to identify prehistoric shifts in the primary producer community structure [18]. A shift from carbonate-dominated to organic-dominated sediment occurred at approximately 5,540 cal yrs BP [18]. The timing of that shift was probably related to the onset of modern hydrological conditions in Florida, as inferred from pollen data [3] and core studies of basal peat layers from the Mississippi Delta [19].

The primary objectives of this study were as follows: 1) assess changes in the major sources of organic matter to the sediments of Lake Harris from its nascent stages in the early Holocene to the present, and 2) determine the environmental and hydrological changes that drove the shifts in organic matter source. We employed a suite of geochemical variables in a complete Holocene sediment record from Lake Harris to evaluate these objectives quantitatively. The concentrations, ratios and stable isotope compositions (δ^13^C, δ^15^N) of sediment total organic carbon (TOC), total nitrogen (TN), and select biomarker hydrocarbons, were used as proxies for the sources of organic matter in the sediment. Geochemical analyses, including concentrations of carbonate (CaCO_3_), biogenic silica (BioSi), and total organic carbon (TOC), were used as proxies for hydrological changes, such as increases or decreases in groundwater input, water table elevation and lake stage. We hypothesized that the predominant sources of organic matter (i.e. allochthonous vs. autochthonous [macrophytes vs. algae]) to the lake’s sediments were controlled by regional hydrologic and environmental conditions that occurred as the Earth’s climate shifted through the Holocene, and human settlement expanded in the 20^th^ century. Specifically, climate transitions included: 1) a steady rise in relative sea level in the Gulf of Mexico that began in the early Holocene and abruptly slowed ~7,000 cal yrs BP [2, 19], wetland formation and expansion in Florida ca. 5,000 cal yrs BP [3, 20] and the onset of the modern El Niño cycle ~3,000 cal yrs BP [21]. The sediment record in Lake Harris shows a response to each of these Holocene climate transitions, and the record from the last ~70–80 years reflects rapid cultural eutrophication.

### Study site

**Fig 1:**
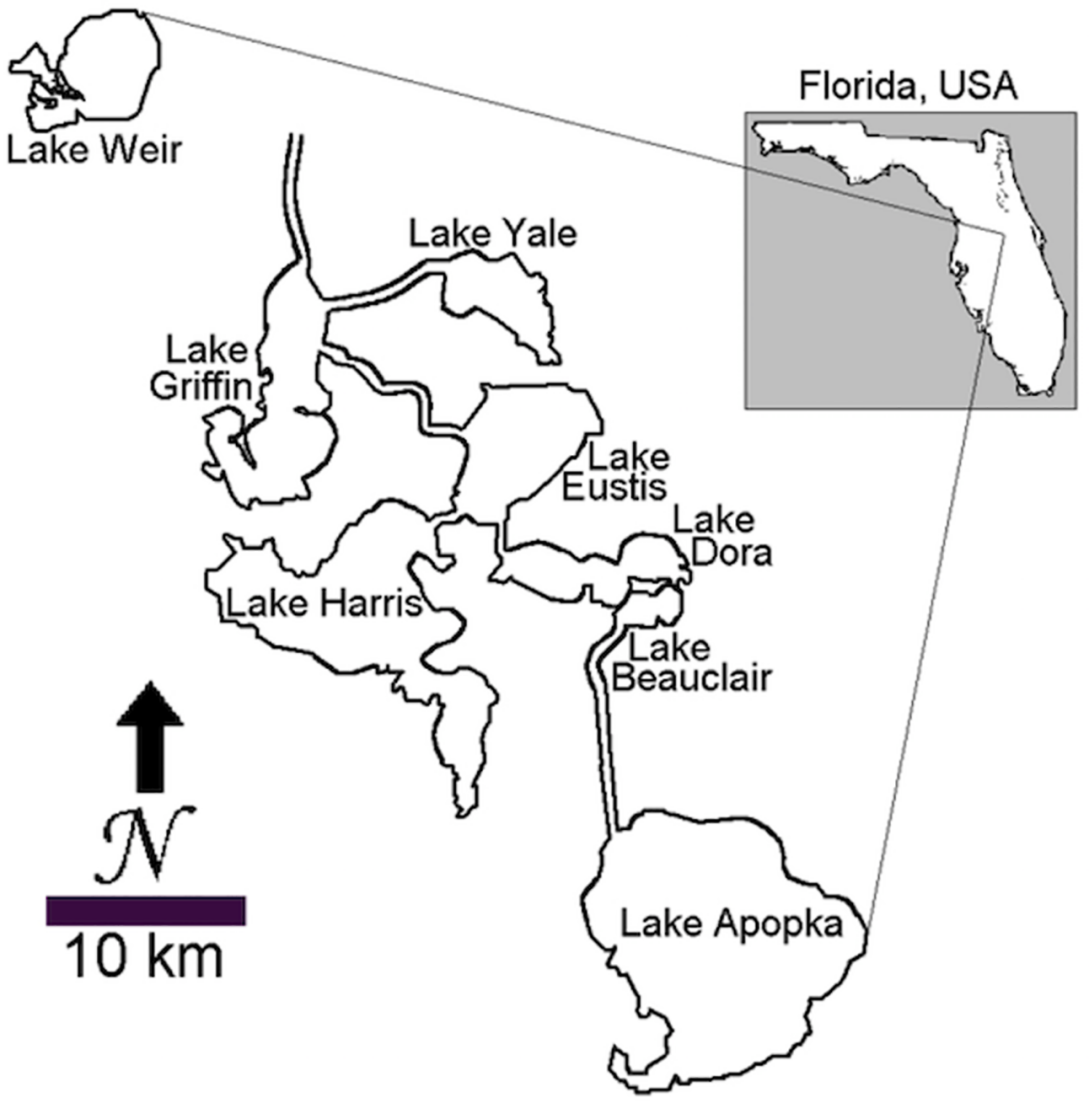
Location of Lake Harris within the Harris Chain of lakes; 28°46′4″N 81°48′57″W. Reproduced from [23] with permission from Springer, license number 4111351386595.

Lake Harris is a shallow (mean depth = 3.5m), productive lake (mean annual chlorophyll a = 57 μg L^-1^, total phosphorus = 38 μg L^-1^, total nitrogen = 1707 μg L^-1^), with a surface area of ~75 km^2^ [22]. It is part of the Harris Chain of Lakes and is located in the Upper Ocklawaha River Basin, Central Florida, USA (Fig 1). Hydrologic flow through some water bodies in the Chain of Lakes begins at spring-fed, hypereutrophic Lake Apopka, which discharges through four other lakes and ultimately into the Ocklawaha River [23]. Three other lakes in the Chain, including Lake Harris, receive minimal or no flow from Lake Apopka and are considered mesotrophic to eutrophic. The hydraulic retention time for Lake Harris is relatively short (2.9 years), but can vary widely between periods of high versus low flow [22]. Most of the lakes in the Harris Chain receive surface and groundwater inputs that pass through organic-rich mineral soils, and thus are considered to be naturally productive and alkaline water bodies [22]. Geochemical analyses on the same 5.9-m sediment record from Lake Harris used in this study, however, indicated the lake was on a trajectory towards increasing oligotrophication throughout the Holocene [18]. This trajectory was interrupted abruptly after ca. AD 1940, when the human population in the region increased six-fold and the lake veered toward the highly productive end of the trophic state spectrum [18].

## Methods

### Sediment sampling

Lake Harris is a public resource, with free access, thus no permitting was required for core collection. Furthermore, no endangered species were involved in this study. We sampled sediment from the 5.9-m core retrieved by [18]. The upper portion of the core was measured for ^210^Pb activity, and the CRS model was used to calculate dates. A date of ~1911 was determined for sediments at 44 cm depth. The remaining portion of the core was AMS-^14^C dated using four charcoal and three bulk sediment samples. Radiocarbon dates were calibrated using INTCAL13. A date on charcoal near the core base had a range of 10500–10250 cal yrs BP, and linear regression of calibrated age versus depth indicated a near-constant sedimentation rate (R^2^ = 0.98). For details of the chronology, see [18].

The core was split in half lengthwise and one half was sampled at 4-cm intervals for geochemical analyses. Wet subsamples were frozen, freeze-dried, and then ground and homogenized for analyses. CaCO_3_, biogenic silica [BioSi] (both diatom- and sponge-spicule-derived), TN, and percent organic matter (%OM) analysis methods are presented in [18]. TOC was measured as the difference between total carbon, measured on a Carlo Erba NA1500 CNS elemental analyzer, and inorganic (carbonate) carbon, measured on a UIC-Coulometrics coulometer, coupled with an Auto-Mate automated carbonate preparation device (AutoMateFX.com). Dried sediment for δ^15^N and δ^13^C_TOC_ analyses was pretreated with 1N HCl to remove inorganic carbon, and then combusted in a Carlo Erba NA1500 CNS elemental analyzer interfaced with a Thermo Scientific Delta V Advantage isotope ratio mass spectrometer. Carbon isotopic compositions were normalized to the VPDB scale and nitrogen isotope values are relative to air. All isotope results are reported in standard delta notation:

$$\delta =\frac{{\text{R}}_{\text{x}}-{\text{R}}_{\text{std}}}{{\text{R}}_{\text{std}}}*1000$$ (1)

### Lipid extraction and quantification

Lipids were extracted from 1-2g of freeze-dried sediment with an Accelerated Solvent Extractor ASE200 (Dionex), using 2:1 (v/v) dichloromethane (DCM):methanol through three extraction cycles at 10.3 MPa (1500 psi) and 100°C. Total lipid extracts (TLE) were concentrated under a gentle stream of nitrogen, and the neutral lipid fraction was obtained after base saponification of the TLE. Neutral lipids were further separated, based on polarity, into compound classes by column chromatography, using 5% deactivated silica gel, according to methods modified from [24]. Hydrocarbons were eluted from the silica gel column with 4.5 mL of 9:1 Hexane:DCM, and saturated hydrocarbons were separated from alkenes on 5% Ag-impregnated silica gel (w/w) with 4 mL of hexane and ethyl acetate, respectively. Branched and cyclic saturated hydrocarbons were isolated from *n*-alkanes with triple urea adduction.

Alkane concentrations were measured and identified on a Thermo Scientific Trace 1310 gas chromatograph with a Supelco Equity 5 column, interfaced to a Thermo Scientific TSQ 8000 triple quadrupole mass spectrometer with electron ionization. The inlet was operated in splitless mode at 280°C. The column flow rate was set to 2.0 mL min^-1^ and the oven was programmed to an initial temperature of 60°C and held for 1 minute, then ramped to 140°C at 15°C min^-1^, and to 320°C at 4°C min^-1^ and held for 25 minutes. Quantification was based on the calibration curves generated from the peak areas of external standards (C_7_-C_40_) with concentrations ranging from 5 to 250 μg mL^-1^. Concentration measurements were reproducible within ±10%.

### Compound-specific isotope measurements on *n*-alkanes

Compound-specific carbon isotope values for *n*-alkanes were measured on an Agilent 6890 GC connected to a Thermo Scientific Delta V Plus IRMS interfaced with a GC-C-III combustion system. The GC flow rate was set to 2.0 mL min^-1^ and the oven was programmed as follows: 60°C for 1 minute, then increased at a rate of 6°C min^-1^ to 320°C, and held for 20 minutes. Compounds were combusted over a nickel/platinum/copper wire with O_2_ at 960°C. The isotope ratios of carbon in CO_2_ were measured and normalized to the VPDB scale using the Uncertainty Calculator [25] and are reported in standard delta notation as above. Standard errors of the mean (SE) were calculated using the Uncertainty Calculator, which yielded a 1σ SE value of ± 0.39‰ [25].

## Results

### Bulk geochemistry and isotopic composition

**Fig 2:**
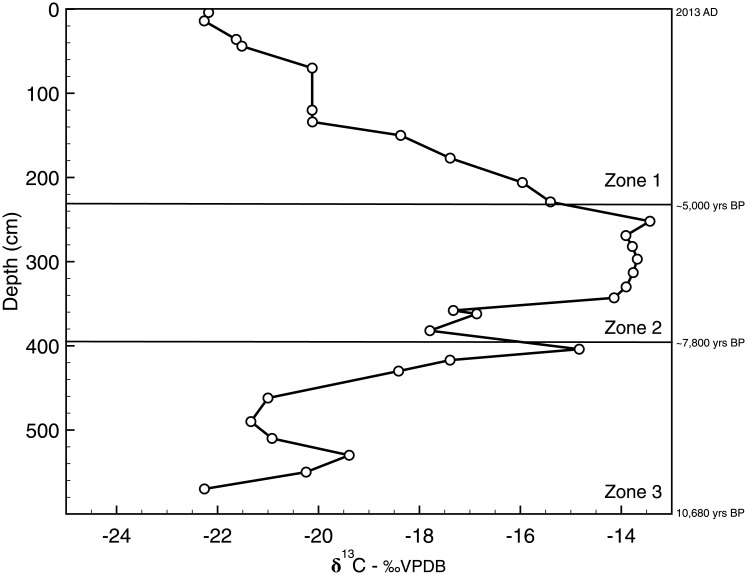
TOC isotope values (δ^13^C_TOC_) versus depth in the Lake Harris core. The three zones in the core are delineated by major shifts in δ^13^C_TOC_ (shown in the figure as solid horizontal lines). Zone 3 is from 590 to 403 cm, zone 2 is from 402 to 241 cm, and zone 1 extends from 240 cm to the core surface.

The core was divided into three zones based on shifts in δ^13^C_TOC_: zone 3 spanned from the bottom of the core to 403 cm (~10,000–7,800 cal yrs BP), zone 2 was from 402 to 241 cm (~7,800–5,000 cal yrs BP), and zone 1 encompassed from 240 cm depth to the surface (~5,000 cal yrs BP-AD 2013) (Fig 2). Zone 3 was marked by an increase in δ^13^C_TOC_ values upcore, from a minimum of -24.29‰ to a maximum of -15.78‰. Bulk carbon isotope values remained relatively stable in zone 2 (mean -14.79‰), before decreasing sharply in zone 1 from -13.27‰ to a minimum value of -22.18‰ at the surface.

**Fig 3:**
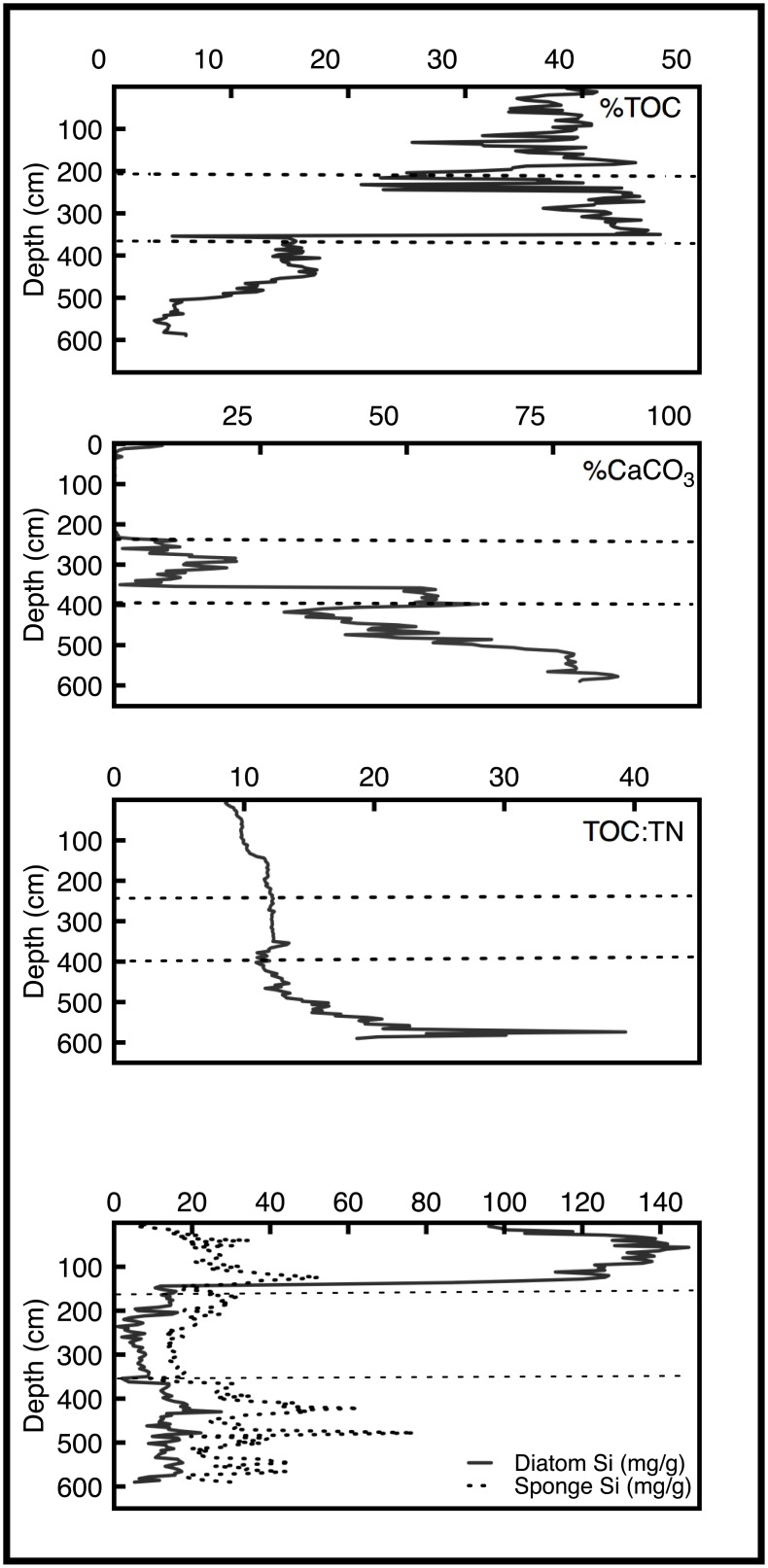
Bulk geochemical variability in the Lake Harris core. From the top panel to the bottom: percent total organic carbon, percent calcium carbonate, molecular total organic carbon to total nitrogen, and diatom and sponge derived silica concentrations.

**Fig 4:**
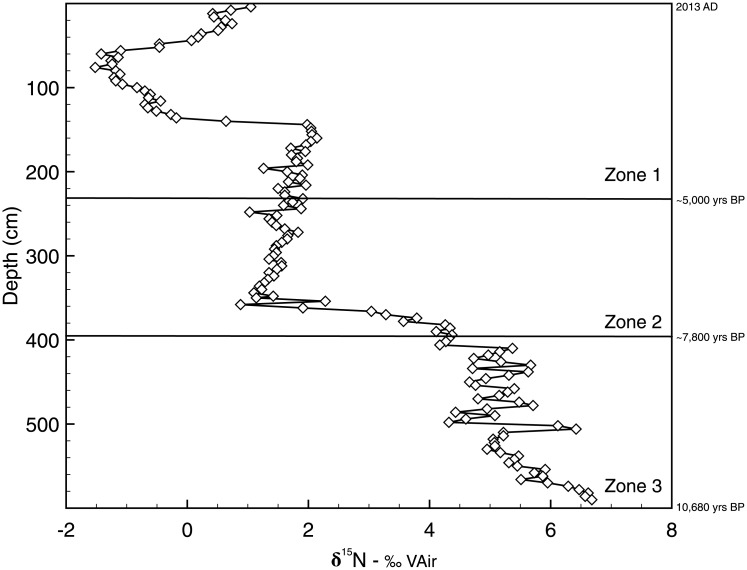
Nitrogen isotope values (δ^15^N) versus depth in the Lake Harris core. Shifts in δ^15^N in the Lake Harris core. Core zones are delineated with solid horizontal lines, and labeled accordingly.

Organic carbon percentages remained below 15% throughout zone 3, while %CaCO_3_ decreased from a maximum value of 86% at 579 cm depth (~10,000 cal yrs BP), to just under 30% at 418 cm (~8,000 cal yrs BP), with an average value of ~60% throughout zone 3 (Fig 3). Values of %TOC and %CaCO_3_ changed markedly at ~350 cm depth (~6,500 cal yrs BP) in zone 2: organic carbon increased from 2.7% to 38.1% and carbonate dropped to 1% and never increased above 25% in zone 2. In zone 1, %TOC remained at elevated concentrations and varied about its mean of 21.3%. Carbonate values in zone 1 never exceeded 11% from 240 cm (~5,000 cal yrs BP) to the surface of the core and displayed an average value of 0.6%. δ^15^N displayed a maximum value of 6.70‰ at the base of the core, in zone 3, but values declined upward, to about 4.00‰ at the top of the zone (Fig 4). Thereafter, values declined rapidly in zone 2, reaching a low of 1.10‰ at ~340 cm. This was followed by a slow increase in δ^15^N values to 2.14 at 160 cm (zone 1), followed by an abrupt decline to a minimum of -1.52‰ at 75 cm core depth. Values then increased to 1.05‰ at the top of the core.

In zone 3, TOC:TN values reached a maximum of 39.3 at 574 cm (~10,000 cal yrs BP), before decreasing rapidly in zone 2 to a minimum value of 10.9. TOC:TN values continued to decrease slowly from zone 2 to zone 1 and reached a minimum value of 8.6 at 8 cm depth (ca. AD 2009) (Fig 3).

Biogenic silica, both diatom- and sponge-derived, displayed considerable variability throughout all zones. Of note was an exponential increase in diatom silica at 144 cm (~2,600 cal yrs BP), where the concentration increased from 12.0 to 52.0 mg g^-1^. These values continued to rise in zone 1, reaching a maximum value of 147.3 mg g^-1^ at 52 cm (~490 cal yrs BP) (Fig 3).

### Concentrations and isotopic compositions of hydrocarbons

**Fig 5:**
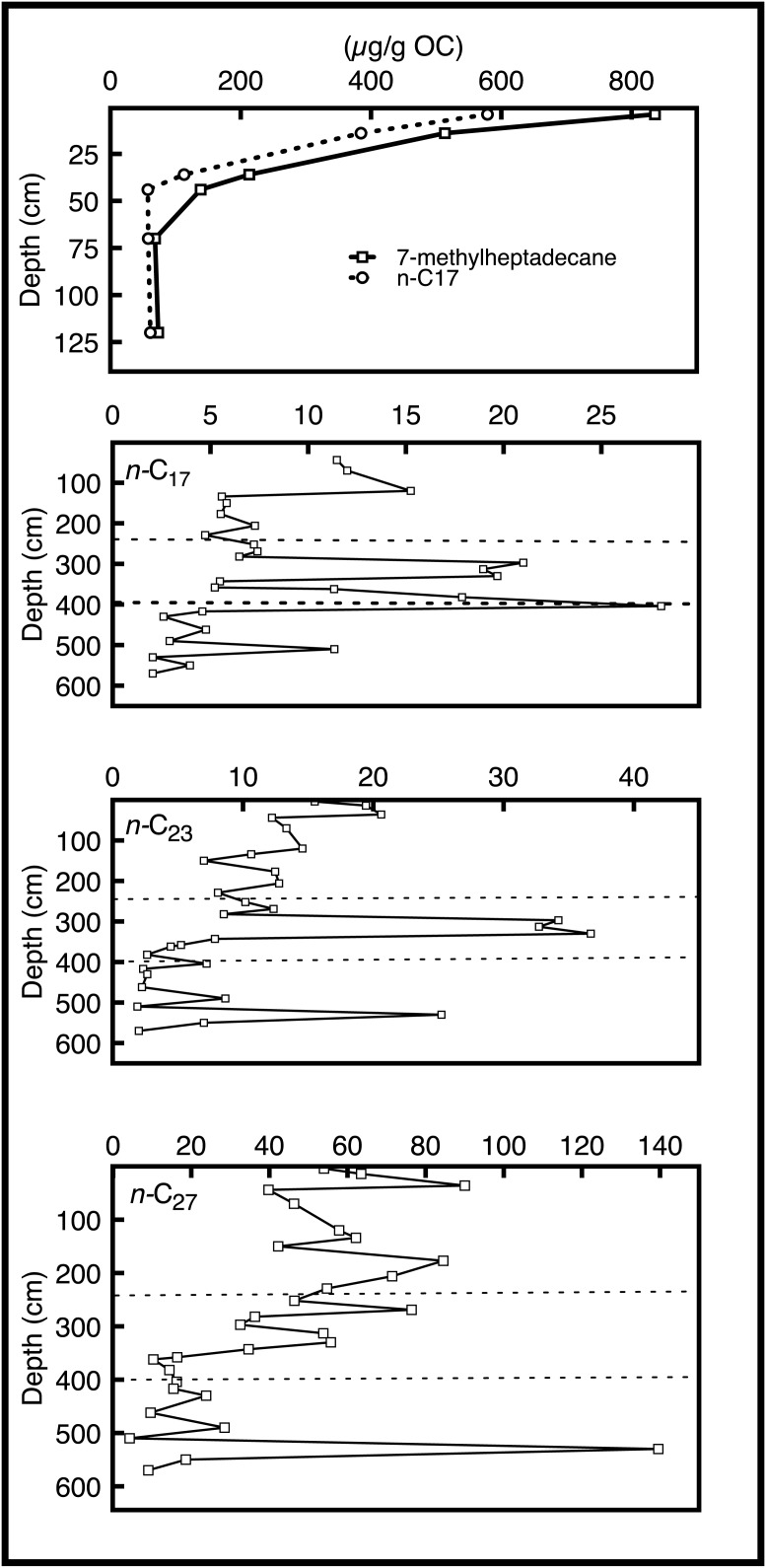
*n*-alkanes. Select *n*-alkane chain length abundances versus depth in the Lake Harris core. Dashed lines delineate core zones 1–3.

Concentrations for select *n*-alkane chain lengths are displayed in Fig 5. Mean *n*-alkane concentrations for these chain lengths are displayed in Table 1. The *n*-alkane concentrations were dominated by long-chain alkanes (i.e. >*n*-C_25_). The most abundant *n*-alkane in our record was *n*-C_27_, with an average concentration of 36.0 μg g^-1^ OC throughout the entire core. We calculated the average chain length (ACL) for all samples using the Eglinton and Hamilton [26] equation:

$$ACL\;=\;\frac{{25C}_{25}+27C_{27}+29C_{29}+31C_{31}+33C_{33}+35C_{35}}{C_{25}+C_{27}+C_{29}+C_{31}+C_{33}+C_{35}}$$ (2)

**Table 1:** *n*-alkane homologues.

*n*-alkane	C_17_ μg g^-1^ OC	C_23_ μg g^-1^ OC	C_27_ μg g^-1^ OC	C_29_ μg g^-1^ OC	C_35_ μg g^-1^ OC
Average	34.1	10.0	36.1	30.3	7.0
Max	482.6	32.5	120.0	79.7	50.5
Min	0.0	0.8	1.7	1.7	0.0
Range	482.6	31.7	118.4	78.0	50.5
Zone 1 Average	81.4	11.2	51.0	40.2	8.1
Zone 2 Average	10.6	12.5	29.2	26.3	4.6
Zone 3 Average	1.2	5.0	25.2	22.3	8.8

Concentrations (average, maxima, minima, and ranges) for the five *n-*alkane homologues used for organic matter source interpretation. Zones are delineated by δ^13^C_TOC_ values.

There was low variability in ACL throughout δ^13^C_TOC_ zones 1 and 3 (ranges = 1.2 and 1.1, respectively), however ACL values in zone 2 vary by 2.8, with a high of 29.7 and a low of 26.9 (Table 2).

**Table 2:** Proxies for organic matter source.

Depth (cm)	ACL	TAR_HC_	P_aq_	Age	Dating Method
4	28.72	0.15	0.47	2012	^210^Pb-AD
14	29.67	0.44	0.29	1998	^210^Pb-AD
36	29.27	2.56	0.30	1943	^210^Pb-AD
44	28.99	6.62	0.38	1912	^210^Pb-AD
70	29.17	6.93	0.30	900	C14-yrs BP
120	29.05	7.02	0.34	1920	C14-yrs BP
134	28.44	28.86	0.31	2400	C14-yrs BP
150	29.38	19.48	0.25	2780	C14-yrs BP
177	29.31	44.10	0.24	3570	C14-yrs BP
206	29.12	19.48	0.29	4770	C14-yrs BP
229	29.25	32.83	0.23	4840	C14-yrs BP
252	29.27	15.01	0.27	5200	C14-yrs BP
269	29.29	26.94	0.21	5450	C14-yrs BP
282	29.48	13.61	0.24	5620	C14-yrs BP
297	27.50	3.32	0.61	5840	C14-yrs BP
313	29.06	7.61	0.42	6050	C14-yrs BP
330	26.92	3.12	0.81	6250	C14-yrs BP
343	29.50	13.76	0.24	6430	C14-yrs BP
358	29.53	14.86	0.22	6740	C14-yrs BP
362	29.73	1.57	0.21	6830	C14-yrs BP
382	28.71	2.08	0.22	7770	C14-yrs BP
404	29.64	1.72	0.30	7850	C14-yrs BP
417	30.15	13.94	0.12	8020	C14-yrs BP
430	29.85	74.75	0.13	8170	C14-yrs BP
462	29.43	4.80	0.20	8480	C14-yrs BP
490	29.29	45.66	0.29	8830	C14-yrs BP
510	29.45	1.46	0.34	9300	C14-yrs BP
530	29.97	51.22	0.32	9470	C14-yrs BP
550	29.03	11.58	0.37	9830	C14-yrs BP
570	29.77	n/a	0.30	9900	C14-yrs BP

Average chain length (ACL) and the ratio of submerged to emergent vegetation (Paq) in the Lake Harris core. Dates determined by the ^210^Pb method are in Anno Domini (AD) and carbon-14 dated layers are in calibrated radiocarbon years before present (cal yrs BP).

Alkane concentrations showed differences across zones in the Lake Harris core (Fig 5). We subdivided alkanes into groups based upon their most probable source(s): cyanobacterial (7-methylheptadecane and diploptene), algal (*n*-C_17_), emergent/submerged macrophytes (*n*-C_23_), woody terrestrial vegetation (*n*-C_27_ and *n*-C_29_), and mixed woody terrestrial/C_4_ grasses (*n*-C_35_). We then related changes in these organic matter sources to regional environmental changes. In broadest terms, the following three zones were discernible based on relative alkane concentrations: zone 3) dominance of terrestrial biomarkers throughout the lower section of the core (below 390 cm), with a concentration maximum at 530 cm (~9,500 cal yrs BP), zone 2) a rapid increase in submerged macrophyte biomarker concentrations at 390 cm (~7,800 cal yrs BP), which persisted until 330 cm (~6,250 cal yrs BP), and zone 1) proliferation of algal and cyanobacterial biomarkers in the top 40 cm of the core (AD 1940 to present).

Specifically, zone 3 was dominated by terrestrial alkane inputs, with *n*-C_27_ and *n*-C_29_ concentrations ~5x that of *n*-C_23_ and ~20x that of *n*-C_17_. P_aq_ values also indicate primarily terrestrial inputs in this zone. A sudden increase in concentrations of *n*-C_23_, *n*-C_27_, *n*-C_29_, and *n*-C_35_ occurs at 530 cm (~9,500 cal yrs BP). Both terrestrial hydrocarbon biomarkers, i.e. *n*-C_27_ and *n*-C_29_, attain maximum values at this depth.

In zone 2, the average concentration of *n*-C_17_ decreases to about 12% of its previous mean value and the terrestrial alkane concentrations are approximately halved, although they remain the most abundant chain lengths in this zone. Of note is an increase in *n*-C_23_ concentrations from 6.2 μg g^-1^ OC at 340 cm (~6,400 cal yrs BP) to 32.5 μg g^-1^ OC at 330 cm, which then dropped back down to 6.9 μg g^-1^ OC at 280 cm (~5,600 cal yrs BP). A similar, albeit smaller increase in *n*-C_17_ concentrations also occurred during this interval (Fig 5).

We evaluated the relative contributions of submerged and floating aquatic macrophytes to emergent terrestrial vegetation using the P_aq_ proxy [27], described by the formula (C_23_ + C_25_)/(C_23_ + C_25_ + C_29_ + C_31_). P_aq_ values from 330 cm (~6,250 cal yrs BP) to 290 cm (~5,700 cal yrs BP) ranged from 0.42 to 0.81, indicative of a freshwater, submerged vegetation source. This is the only section of the Lake Harris core that has P_aq_ values that fall in the range of submerged vegetation.

Below 120 cm (~1,900 cal yrs BP) in zone 1, concentrations of *n*-C_17_ and 7-methylheptadecane were < 5 μg g^-1^ OC. In this lower section of zone 1, terrestrial biomarkers *n*-C_27_ and *n*-C_29_ were the most abundant hydrocarbons, with average concentrations of 52.3 and 43.3 μg g^-1^ OC, respectively. In the upper 40 cm of zone 1 (AD 1940 to present), however, there was a ~50-fold increase in the concentration of *n*-C_17_ and a ~40-fold increase in 7-methylheptadecane concentration.

Meyers [13] developed a hydrocarbon-based proxy, known as the terrestrial to aquatic ratio (TAR_HC_), to distinguish between autochthonous and allochthonous sources of organic matter in sediments. TAR_HC_ results matched our comparative analyses of single-chain-length alkane concentrations (Table 2). Values <1 reflect dominance of aquatic hydrocarbons, and are only observed in the top 15 cm of our core, i.e. after AD 1998. TAR_HC_ are highly variable, but average 16.4, which indicates that contributions of carbon from terrestrial plants dominated the record. The maximum TAR_HC_ (74.8) was found at 530 cm (~9,500 cal yrs BP), whereas values <4 occur at 510 (~9,300 cal yrs BP), 400–360 (~7,800–6,750 cal yrs BP), 330–290 (~6,250–5,700 cal yrs BP), and 35 cm (AD 1945).

**Fig 6:**
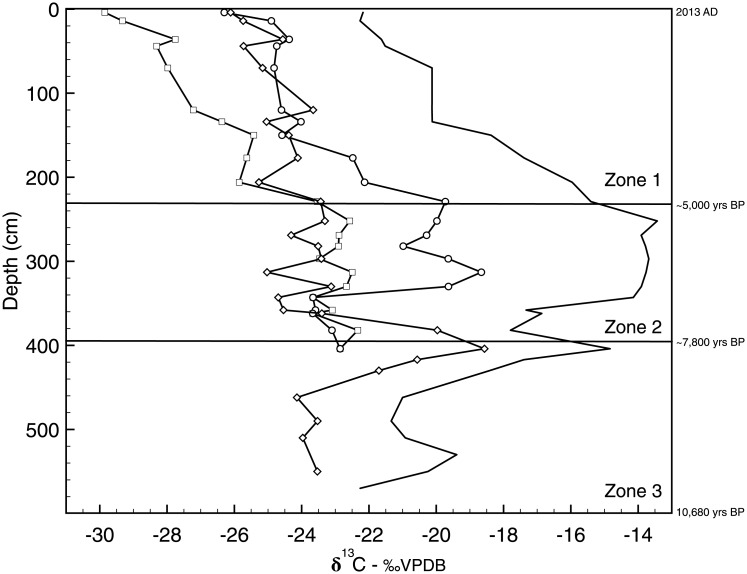
*n*-alkane isotopic variability. Carbon isotope variability in select *n*-alkane chain lengths in the Lake Harris core.

Isotopic compositions of selected *n*-alkane chain lengths are shown in Fig 6. Based upon *n*-alkane concentrations and organic matter source assignments, we interpreted isotope values for the following hydrocarbons in this study: 7-methylheptadecane, *n*-C_17_, *n*-C_23_, *n*-C_27_, and *n*-C_29_. Reliable carbon isotope ratios could not be measured on *n*-C_17_ and *n*-C_23_ below 415 cm depth.

The carbon isotopes of 7-methylheptadecane had the highest average value for any biomarker in our core. From 45 cm to the sediment surface, the δ^13^C values of this branched alkane averaged -21.30‰, and varied by only 0.7‰ across those depths. The δ^13^C signature of *n*-C_17_ decreased progressively across all zones from a maximum value of -22.3‰ at 380 cm depth, to a minimum value of -29.9‰ at the top of the core. From 400 to 313 cm (~7,800–6,050 cal yrs BP) (zone 2), δ^13^C of *n*-C_23_ increased from -22.8‰ to -18.7‰, then stabilized at a mean value of -19.9‰ throughout the rest of zone 2. In zone 1, carbon isotope values of *n*-C_23_ decreased from a maximum of -19.73‰ to a minimum value of -26.30‰ at 4 cm depth (AD 2011). From the bottom of the core to 400 cm (the base of zone 2, ~7,800 cal yrs BP), δ^13^C values of vascular plant alkanes *n*-C_27_ and *n*-C_29_ both increase in a pattern that follows the increase in δ^13^C_TOC_. Carbon isotope values then decreased rapidly at 362 cm (~6,820 cal yrs BP) for *n*-C_27_, and at 400 cm for *n*-C_29_. These chain lengths returned relatively constant δ^13^C values in core zone 1, with average values of 24.8‰ and 28.0‰ for *n*-C_27_ and *n*-C_29_, respectively (Fig 6).

## Discussion

Shifts in values of geochemical variables in the sediments of Lake Harris coincide with major environmental changes that occurred in the region during the Holocene. Hydrocarbon biomarker data indicate a series of hydrological shifts that resulted following Northern Hemisphere deglaciation. These shifts in Lake Harris are temporally linked to records from other lakes in north and central Florida [3, 27, 28]. Broadly speaking, Lake Harris evolved from a marsh-like system in the early Holocene, to a shallow lake in the middle Holocene, and then deepened to its modern state after ~2,800 years BP. Carbon isotope values of the autochthonous and allochthonous organic matter pools are proxies for these environmental changes and reflect multiple sources of inorganic carbon utilization by the primary producer communities.

### Geochemical biomarkers

Biological sources of alkanes are described in numerous publications as reviewed in Bianchi and Canuel [11]. The *n*-C_27/29_ homologues of *n*-alkanes represent organic matter sourced from woody terrigenous plants [29]. C_4_ graminoids contain *n*-C_35_ alkanes in their leaves at concentrations an order of magnitude greater than those in woody C_3_ angiosperms [30, 31]. Submerged and emergent macrophytes typically produce alkanes with chain lengths of *n*-C_21/23_ [27], and algae synthesize alkanes with a predominant chain length of *n*-C_17_ [29]. Additionally, branched alkanes 7- and 8-methylheptadecane are produced exclusively by cyanobacteria and account for 90% of their total branched alkanes [32]. Although hydrocarbon biomarkers are source-specific, they represent only a small fraction of the total carbon content of bacteria and plants. Biomarker data gleaned from this study complement the bulk sediment carbon analyses that characterize much larger fractions of the organic matter.

The earliest portion of the core (zone 3) is dominated by *n*-alkanes derived from terrestrial and, to a lesser extent, macrophyte sources, as well as abundant sponge-spicule-derived biogenic silica. At a depth of 550 cm in the core, approximately 800 years after the lake began to fill with water in the early Holocene (10,600 cal yrs BP), there was a pulse of *n*-C_23_, *n*-C_27_, and *n*-C_29_ to the sediments (Fig 5). This occurred shortly after TOC:TN values increased from 24 to 39 at 574 cm (~10,000 cal yrs BP). TOC:TN values continued to increase toward maximum values, in agreement with the high abundances of terrestrial biomarkers. During this earliest stage in the lake’s evolution, it was a shallow, low-productivity, marsh-like system that received abundant vascular plant input from littoral communities (e.g. *Taxodium* spp., *Salix* spp. and *Cephalanthus occidentalis*), but also supported macrophyte growth. The temporal correlation between the TOC:TN, sponge spicules, and terrestrial hydrocarbon biomarkers is in strong agreement with palynological [33] and diatom-based paleolimnological reconstructions [34], all of which describe central Florida lakes as low-nutrient, shallow-water systems during the early Holocene.

Zone 2 is highlighted by localized maxima of algae and macrophyte biomarkers (Fig 5). The increase in *n*-C_23_ begins at 358 cm (~6,700 cal yrs BP), which corresponds to a shift from predominantly CaCO_3_ sediments to organic-rich sediments without measurable carbonate by 232 cm (~5,000 cal yrs BP) (Fig 3). The decrease in carbonate content in lake sediments coincided with decreases in proxy variables indicative of extensive macrophyte coverage in Lake Harris, e.g. sponge-derived biogenic silica, ACL values < 27, and average P_aq_ values of 0.61 (Fig 3). Kenney et al. (2016) correlated these geochemical changes with the proliferation of *Pinus* pollen in Florida and the onset of wetter conditions in upland areas during the middle Holocene [3]. We infer that during this interval, hydrologic input to Lake Harris shifted from a predominantly groundwater source, early in zone 2, to primarily direct rainfall and runoff, later in zone 2.

Lake Panasoffkee, in central Florida, is a modern analogue for Lake Harris during the early to middle Holocene. Currently, Lake Panasoffkee receives substantial groundwater inputs and is a hard-water, macrophyte-dominated system. TOC:TN values (14–22) of submerged aquatic vegetation (SAV) in Lake Panasoffkee [35] are only slightly greater than the average TOC:TN value in zone 2 of the Lake Harris core (12.1). From this, we infer that sediment organic carbon in Lake Harris from ~8,000–5,000 cal yrs BP was sourced from a mixture of macrophytes and low-TOC:TN algae and periphyton. This inference is further supported by vegetation studies from shallow, low-nutrient lakes that have primary producer communities dominated by SAV [36]. The *n*-alkane biomarker concentrations, with maxima of *n*-C_17_ (at ~400 and 330 cm; ~7,800 and 6,250 cal yrs BP, respectively) and *n*-C_23_ (at ~330 cm; ~6,250 cal yrs BP), the sponge-derived silica concentrations, and the TOC:TN data from modern SAV, periphyton, and algae, coincide with one another in zone 2 of our core. Together they indicate a primarily groundwater-fed system with abundant submerged and emergent macrophyte populations.

**Fig 7:**
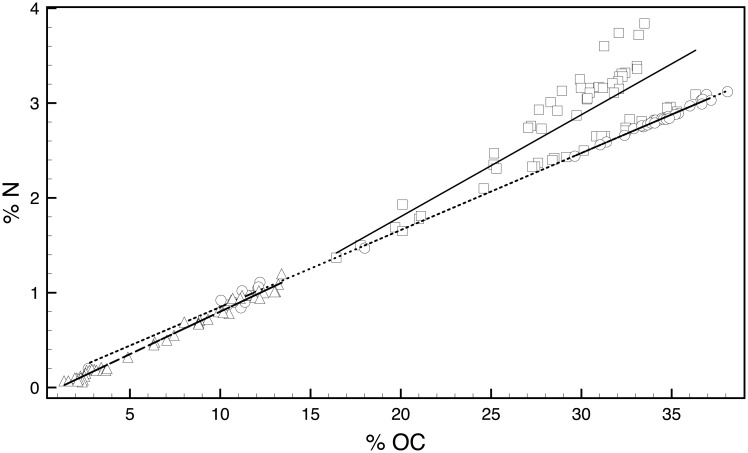
Plot of total organic carbon percent versus total nitrogen. The plot is divided according to core zone: zone 1, squares with solid trend line (R^2^ = 0.72, b = -0.72), zone 2, circles with dashed trend line (R^2^ = 0.99, b = 0.03), and zone 3, triangles with dotted trend line (R^2^ = 0.99, b = -0.09). For all samples R^2^ = 0.95 and b = -0.09).

The largest shift in any of the hydrocarbon concentrations occurred in zone 1. Between about 240 cm (~5,000 cal yrs BP) and 40 cm (AD 1940), concentrations of biomarkers for the three major organic matter sources are fairly evenly distributed. Indeed, within that sediment depth range, combined algal and macrophyte *n*-alkane concentrations equate to approximately half of the terrestrial *n*-alkane concentrations. Terrestrial (*n*-C_27_) and macrophyte (*n*-C_23_) biomarker concentrations fluctuate between high and low values, but display no discernible trend across zone 1. In the top ~40 cm of the core, ca. AD 1940 to present, however, *n*-C_17_ and 7-methylheptadecane increased by about 50-fold and 40-fold, respectively. This rapid increase in algal and cyanobacterial biomarkers corresponds with the timing of an increase in trophic status of many other Florida lakes and a six-fold population increase in the counties surrounding the Harris Chain of Lakes after about AD 1940 [22]. The 7-methylheptadecane concentrations, in particular, are indicative of a highly eutrophic, possibly N-limited lake that supports cyanobacteria proliferation [37]. The decreasing trends in δ^15^N values (Fig 4), that approach 0‰, further support the relative increase in N-fixing cyanobacteria during the top of zone 1 [38]. In some ecosystems, the presence of inorganic nitrogen (e.g. ammonium) sorbed to clays can alter the δ^15^N signal. This is not applicable in Lake because its sediments lack clay and have high organic matter content. Furthermore, a plot of TOC vs TN is strongly coupled (r^2^ = 0.95), and the intercept of the regression line is nearly 0 (Fig 7). If inorganic nitrogen contributed to the sediment N pool, the intercept of Fig 7 would be >0. It is possible that the isotopic signature of organic matter is altered, in situ, but this alteration would have been minor and remained consistent throughout the record.

Trends in the biomarker data, however, are not fully supported by the bulk geochemical data. Whereas concentrations of algal and bacterial biomarkers are an order of magnitude greater than concentrations of any terrestrial biomarker throughout the top 40 cm of the core, the TOC:TN values do not display a decrease, as would be expected with an increase in non-vascular plant sources. Instead, TOC:TN values remain relatively stable throughout zone 1, with an average value of 10.2, nevertheless indicative of a primarily algal source [39]. Post-depositional alteration of TOC:TN values occurs in sediment pore waters [11], and decomposition can remove as much as 20% of the organic matter that is buried on the lake bottom [39, 40]. Kenney et al. [41] compared cores collected in 1999 and 2013 at the same location in Lake Harris and detected loss of organic matter from sediments < 50 years old during the ~13-year interval between core collection dates. Because Lake Harris is shallow and polymictic, organic matter can degrade in the water column year-round, which likely results in significant pre-depositional decay. In fact, in Lake Michigan, it was shown that only 6% of the autochthonously produced organic matter reached the sediment surface [42]. Although loss of sinking organic matter to heterotrophic oxidation in a shallow water body such as Lake Harris (3.5 m mean depth) is expected to be considerably less than in a deep lake like Lake Michigan (85 m mean depth), we can assume that some fraction is lost before burial in the Harris sediment. Overall, TOC:TN data are limited in their ability to reflect source information because of selective degradation and organic matter processing in the water column. Additionally, TOC:TN values in zone 1 have the weakest correlation, suggesting multiple N contributions to the lake during this period (Fig 7) [43]. Because the measured TOC:TN value of a sample reflects multiple organic matter sources and post-depositional processes, its utility for source inference is compromised relative to the hydrocarbon biomarker data.

### δ^13^C_TOC_ and hydrocarbon biomarkers

Measures of δ^13^C_TOC_ fluctuate from low values in the earliest part of the core (~10,000–7,800 cal yrs BP) to the greatest values in the middle section (~7,800–5,000 cal yrs BP), and then return to lower values in the most recent section (~5,000 cal yrs BP to present) (Fig 2). This variability reflects, in part, the changing sources of carbon utilized by the primary producer communities. In the early Holocene, Lake Harris began to fill as the water table in the region started to rise in response to deglaciation and sea level rise [44]. Most lakes in Florida began to accumulate sediment at that time, but remained shallow marsh systems for millennia, as precipitation and water table elevations both stayed relatively low during the early Holocene [28, 45, 46]. The carbon isotope values of *n*-C_27_ show a stratigraphic pattern similar to that of δ^13^C_TOC_ throughout zone 3. The δ^13^C values of *n*-C_27_, adjusted for the 4.4‰ depletion during alkane synthesis, plot within the isotopic range δ^13^C_TOC_ values. The environmental reconstructions and carbon isotope data indicate that the major source of organic carbon to the lake sediments in zone 3 was from terrestrial plants that utilized atmospheric CO_2_.

As Florida gradually became wetter during the middle Holocene (between 7,000 and 5,500 cal yrs BP, zone 2 in our record) the sclerophyllous oak and open prairie plant communities were replaced by modern vegetation communities, largely dominated by pine forests, at least in upland sites [3, 47]. The first significant change in δ^13^C_TOC_ values occurred at ~6,500 cal yrs BP (350 cm core depth) and corresponds to this environmental change. The increase in δ^13^C_TOC_ by ~10‰ from zones 3 to 2 implies that a new source of inorganic carbon was being utilized by the primary producers. The average δ^13^C_TOC_ value (14.8‰) is within the expected range of C_4_ vegetation, meaning a shift from C_3_ to C_4_ communities could also be a cause of the δ^13^C_TOC_ increase. This, however, is improbable for two reasons. First, C_4_ plants are adapted to low *p*CO_2_ and arid environments [48], and the middle Holocene was a period of increasing precipitation and relatively high *p*CO_2_ [3, 49]. Second, our *n*-C_35_ record, which is a proxy for graminoid abundance [31], and the record of C_3_/C_4_ changes from nearby Lake Tulane [50], do not indicate an increase in the relative abundance of C_4_ plants during this time interval. It is also possible that the ^13^C enrichment was a consequence of decreased carbon isotope discrimination among phytoplankton under highly eutrophic conditions. This has been observed in several high-productivity lakes in Florida [15, 51], and has been attributed to recent cultural eutrophication in others [14]. If this were the cause of the higher δ^13^C_TOC_ values, then Lake Harris would have been eutrophic during the middle Holocene, but Kenney et al. [18] demonstrated that nutrient accumulation was much lower at that time compared to modern nutrient accumulation rates.

The δ^13^C_TOC_ record in zone 2 reflects a shift to a more ^13^C-enriched carbon source for the in-lake primary producer community. The δ^13^C value of *n*-C_23_ is the probable source for this enrichment, as: 1) this chain length exhibits the highest δ^13^C values across the depth interval 350–250 cm (~6,500–5,150 cal yrs BP), 2) these values, once corrected for the 3.2‰ offset during alkane synthesis, nearly overlap with the TOC isotope data, and 3) the *n*-C_23_ carbon isotope profile tracks the total organic carbon isotope data throughout zone 2. Lake Harris deepened as the modern hydrosphere developed. Algae initially filled this newly created niche. From 430 to 400 cm (~8,200–7,800 cal yrs BP) there was a ~15-fold increase in *n*-C_17_ concentrations. In Lake Griffin, which is part of the Harris Chain of Lakes, a similar mid-Holocene peak in algal abundance was recorded in the form of sedimented cyanotoxins [52]. We speculate that a relatively rapid rise in water levels initially supported phytoplankton populations, which later gave way to the proliferation of slower-growing macrophytes, the latter indicated by a ~30-fold increase in *n*-C_23_ contributions to the sediment at 380 to 330 cm (~7,700–6,250 cal yrs BP).

We propose two possible explanations for the ^13^C enrichment of the TOC. First, high CaCO_3_ concentrations in the sediment from the beginning of zone 3 to 350 cm (~6,500 cal yrs BP) indicate a well-buffered system with high pH (>9). Bicarbonate (HCO_3_^-^) is the major form of DIC at pH >9, with dissolved CO_2_ absent above pH ~8.3, and active uptake of this HCO_3_- would have enriched the phytoplankton and SAV biomass by 8‰ relative to CO_2(aq)_ [53]. This could explain the high δ^13^C values measured in *n*-C_23_. Secondly, Brenner et al. [35] measured modern δ^13^C values in two SAV taxa (*Vallisneria* sp. and *Potamogeton* sp.) from nearby Lake Panasoffkee, and their δ^13^C range (-13.2‰ to -15.5‰) is enough to explain the elevated δ^13^C of the bulk organic matter in zone 2 of Lake Harris. The relatively high δ^13^C values in zone 2 suggest proliferation of *Vallisneria* sp. and *Potamogeton* sp. in Lake Harris during this time period.

From 250 cm core depth (~5,000 cal yrs BP) to the present (AD 2013) (zone 1), the organic matter becomes progressively depleted in ^13^C. This depletion coincides with a second stage of increased precipitation in Florida that occurred between 5,000 and 3,000 cal yrs BP [47]. Pollen-based reconstructions of summer precipitation show persistently wetter conditions after 3,000 cal yrs BP, which were related to the intensification of ENSO [46]. During that period, south Florida transitioned from a wet prairie to swamp forest environment [54]. Farther north, Quillen et al. [46] linked changes in benthic diatom assemblages to an increase in water depth in Lake Annie around the same time that Donders et al. [54] noted ENSO intensification. In nearby Lake Apopka, a shift from dominance of benthic diatoms (>50%) to dominance of planktonic diatoms (>50%) ~2,800 cal yrs BP indicated that the system had increased in depth sufficiently to approximate its modern limnetic state [28]. Geochemical proxies in the Lake Harris core indicate it also achieved its modern water depth around that time. Organic matter abundance stabilized at levels >50% and CaCO_3_ dropped to trace levels after ~4,800 cal yrs BP (Fig 3). Diatom biogenic silica concentrations exceeded sponge-derived silica concentrations at ~2,800 cal yrs BP. Together these sediment variables reflect a transition from a primarily shallow, groundwater-fed lake, to a relatively deep lake, whose depth was controlled by precipitation and/or surface water inputs.

Our *n*-alkane data also support this interpretation. The more negative δ^13^C_TOC_ values in zone 1 results from increased algal contributions to the sediment. The algal proxy *n*-C_17_ has the most depleted δ^13^C values among all *n*-alkanes analyzed, and the transition to more negative values in the bulk isotope data signifies the rising contribution of algae to the organic matter pool. Interestingly, this is the opposite trend observed by Filley et al. [17] in Mud Lake, Marion County, Florida. In that study, the greatest δ^13^C values were recorded in *n*-C_17_. We argue that the photic zone to water column ratio is larger in Mud Lake (mean depth = 1 m) than in Lake Harris (mean depth = 3.5 m), and therefore isotopic discrimination by the primary producers has a greater impact on increasing the δ^13^C_TOC_ value. The dissolved inorganic carbon (DIC) pool in Lake Harris is larger, because it is deeper, thus primary productivity has a smaller impact on the isotopic signature of the DIC pool.

The cyanobacteria biomarker 7-methylheptadecane is the biomarker in highest abundance in the top 35 cm (AD ~1940-present) of the core. It also exhibits the highest average δ^13^C values among all hydrocarbons in zone 1. Despite this, the δ^13^C_TOC_ values do not become more positive up-core, even though greater δ^13^C_TOC_ values would be expected as the lake became more eutrophic and cyanobacterial blooms thrived [14]. The average δ^13^C values of 7-methylheptadecane (-21.3‰) are nearly identical to values measured in the top 30 cm of Mud Lake (-21.9‰) by Filley et al. [17]. Diagenetic alteration of the cyanobacteria signal is unlikely, as these compounds are present in abundant concentrations in our record, and are major components in the surface sediments of other Florida lakes [52, 55]. Our data show that diatom silica accounts for more than 10% of sediment mass in zone 1, so the discrepancy between δ^13^C_TOC_ and the δ^13^C of 7-methylheptadecane is probably a result of high productivity of diatoms and other algae in Lake Harris.

### Conclusions

The evolution of Lake Harris is recorded in the geochemical variables preserved within its sediments. The lake began to fill with water ~10,000 cal yrs BP, when many lakes in Florida began to accumulate sediment in response to wetter conditions caused by deglaciation and eustatic sea level rise. From the bottom of the core to 403 cm depth (~7,800 cal yrs BP), terrestrial carbon inputs dominated the record, with limited input from macrophytes and algae, and the primary carbon source was atmospheric CO_2_. Throughout the early Holocene, Lake Harris was a marsh-like system in a relatively dry, open-prairie environment. A rapid, positive shift in δ^13^C_TOC_ values at 402 cm (~7,800 cal yrs BP), and stabilization of these values at 350 cm (~6,500 cal yrs BP), represents the onset of wetter conditions in Florida. High CaCO_3_ concentrations in the deepest sediments indicate that Lake Harris was fed primarily by groundwater, and isotopic values of *n*-alkanes and bulk organic matter suggest that organic matter was derived primarily from macrophytes that utilize ^13^C-enriched HCO_3_^-^ as their carbon source. Alternatively, higher δ^13^C_TOC_ values after ~7,000 cal yrs BP may have resulted from input of *Vallisneria* sp., with its higher δ^13^C signature. It was during that time, between 7,000 and 5,000 cal yrs BP, that Florida uplands transitioned from dry oak scrub vegetation to pine forests. Above 240 cm core depth (~5,000 cal yrs BP), δ^13^C_TOC_ values begin to decline, CaCO_3_ concentrations in the sediments decrease to below trace levels, and organic carbon concentrations increase. Around 3,000 cal yrs BP the effects of ENSO intensified and many Florida lakes deepened to their current limnetic state. This is observed in Lake Harris ~2,900 cal yrs BP, when diatom biogenic silica concentrations increased from 10 to 120 mg g^-1^. Concentrations of algal and cyanobacteria biomarkers increased by orders of magnitude after about AD 1940 in response to human-induced eutrophication.

## Supporting information

S1 Table Bulk data—Arnold et al. (XLSX)

S2 Table Biomarker data—Arnold et al. (XLSX)
