# Correction: Sediment biomarkers elucidate the Holocene ontogeny of a shallow lake

**DOI:** 10.1371/journal.pone.0203801

**Published:** 2018-09-07

**Authors:** T. E. Arnold, W. F. Kenney, J. H. Curtis, T. S. Bianchi, M. Brenner

Fig 6 legend is not complete. Please see the complete, correct Fig 6 caption here.

**Fig 6:**
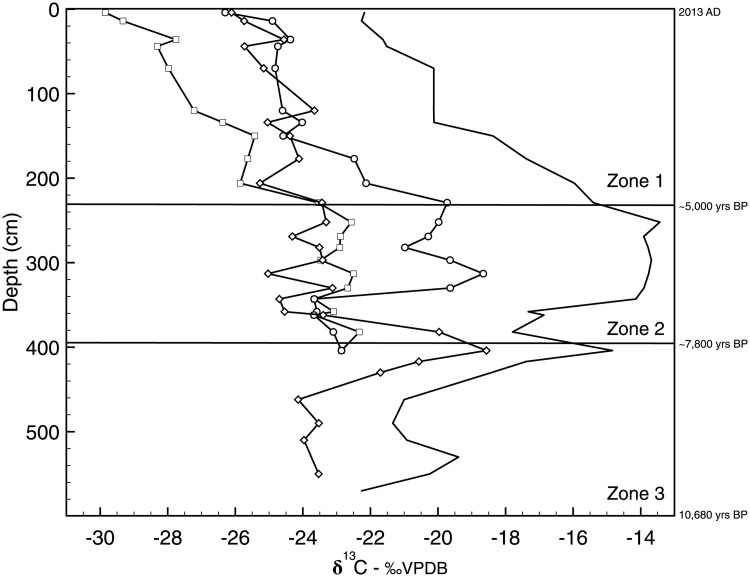
*n*-alkane isotopic variability. Carbon isotope values of TOC (solid line) and select *n*-alkane chain lengths (C_17_, C_23_, C_27_) versus depth in the Lake Harris core. *n*-C_17_ (squares), *n*-C_23_ (circles), and *n*-C_27_ (diamonds).
